# Insights Into monoclonal antibodies and vaccines targeting respiratory viruses

**DOI:** 10.3389/fped.2026.1864969

**Published:** 2026-07-30

**Authors:** Antonella Gambadauro, Giovanna A. Piedimonte, Sara Manti, Giovanni Piedimonte

**Affiliations:** 1 Pediatric Unit, Department of Human Pathology in Adult and Developmental Age “Gaetano Barresi”, University of Messina, Messina, Italy; 2 Columbia College, Columbia University in the City of New York, New York, NY, United States; 3 Departments of Pediatrics, Biochemistry & Molecular Biology, Tulane University, New Orleans, LA, United States

**Keywords:** active immunization, influenza, metapneumovirus, monoclonal antibodies, parainfluenza, passive immunization, respiratory syncytial virus, rhinovirus

## Abstract

**Introduction:**

Viral respiratory infections are a leading cause of morbidity and mortality in children worldwide, with lower respiratory tract infections (LRTIs) a major contributor to hospitalizations and healthcare utilization. Among respiratory pathogens, respiratory syncytial virus (RSV) is the leading cause of severe disease in early life and has driven major advances in preventive strategies. Increasing evidence also suggests that early-life viral infections may influence long-term respiratory outcomes, including recurrent wheezing and asthma.

**Aim of the study:**

This article provides a comprehensive overview of the epidemiological burden, risk factors, and long-term consequences of pediatric viral infections. It also examines RSV as a case study in the evolution of preventive strategies and explores knowledge that can be translated from RSV management to other major respiratory viruses, including influenza, SARS-CoV-2, rhinovirus, human metapneumovirus (HMPV), and parainfluenza.

**Discussion:**

RSV has reshaped pediatric respiratory prevention through the development of monoclonal antibodies and maternal vaccination strategies, enabling a shift from high-risk-based approaches to broader population-level protection. Advances in structural virology have enabled targeting of conserved viral epitopes, thereby improving efficacy and the duration of protection. Similar strategies are being explored for other respiratory viruses. However, antigenic variability, limited pediatric data, and incomplete vaccine effectiveness remain significant barriers.

**Conclusion:**

RSV experience provides a robust foundation for preventing viral respiratory infections. Integrating passive and active immunization strategies, targeting conserved viral structures, and improving global access are essential to reduce the disease burden of pediatric respiratory infections.

## 1 Introduction

Viral respiratory infections are a leading cause of morbidity and mortality in children worldwide, accounting for a substantial share of outpatient visits, hospitalizations, and healthcare utilization (1). Among these, lower respiratory tract infections (LRTIs) remain a major global health concern, particularly in infants and young children, whose immunological vulnerability increases disease severity (2). Respiratory syncytial virus (RSV) alone causes approximately 33 million LRTI cases, 3.6 million hospitalizations, and more than 100,000 deaths annually among children under 5 years of age worldwide (3, 4). Despite advances in supportive care, the burden of pediatric respiratory infections persists, with marked geographic and socioeconomic disparities driven by differences in healthcare access, environmental exposures, and preventive strategies. Most RSV-related deaths occur in low- and middle-income countries, where access to timely supportive care remains limited (5). These inequalities underscore the need for safe and effective prevention strategies. In recent years, attention has increasingly focused on identifying high-risk populations and understanding the long-term consequences of early-life viral infections. A growing body of evidence suggests that severe respiratory infections in infancy may contribute to chronic respiratory morbidity, including recurrent wheezing and asthma, through complex interactions between viral pathogenic mechanisms and host immune responses (2). These observations have reinforced the need for preventive strategies that mitigate disease burden both in the acute phase and over the long term. In this regard, RSV prevention has emerged as an emblematic case study. As the leading cause of hospitalization for respiratory infections in the first year of life, RSV has driven major advances in the development of both passive and active immunization strategies.

Preventive strategies against respiratory viral infections can be broadly categorized into active immunization, which stimulates host immune responses through vaccination, and passive immunization, which provides immediate protection through administration of pathogen- specific antibodies. While first-generation RSV monoclonal antibodies were limited by short half- lives, the need of repeated doses, and restricted indications, recent advances in antibody engineering have allowed the development of next-generation monoclonal antibodies with prolonged half-lives, broader eligibility criteria, and season-long protection after a single administration (6–10). The evolution from first-generation monoclonal antibodies to long-acting agents, together with the introduction of maternal vaccination, marks a transformative shift toward broader and more effective RSV prevention (6–10). Similar approaches are now being explored for other pediatric respiratory pathogens. While maternal vaccination is already established and recommended for infections, such as influenza and severe acute respiratory syndrome coronavirus 2 (SARS-CoV-2), advances in RSV prevention have renewed interest in expanding both passive and active immunization strategies to additional respiratory viruses, including HMPV, rhinovirus, and parainfluenza, where antigenic diversity and safety concerns have limited progress (11–14).

This review aims to (a) provide a comprehensive overview of the burden of viral respiratory infections in children, focusing on risk factors and long-term outcomes, and (b) examine RSV as a model pathogen for innovation in prevention. It also explores how lessons learned from RSV can strengthen preventive strategies against other major pediatric respiratory viruses, including influenza and SARS-CoV-2. To this end, we conducted a structured literature search in PubMed (MEDLINE) through March 2026, using combinations of keywords and MeSH terms related to viral respiratory infections, pediatric populations, epidemiology, risk factors, long-term outcomes, and preventive strategies. Priority was given to systematic reviews, meta-analyses, randomized controlled trials, and large observational studies, which were complemented by manual screening of reference lists. Only articles published in English were included, and the evidence was synthesized using a narrative approach.

## 2 Burden of viral respiratory infections in children

### 2.1 Epidemiology and clinical impact

Viral respiratory infections are among the most common causes of disease across age groups worldwide and impose a substantial burden on healthcare systems. LRTIs are the leading infectious cause of death worldwide and contribute significantly to morbidity, hospitalizations, and healthcare utilization in children. In 2023, LRTIs ranked as the fourth-leading cause of death globally across all age groups and the second-leading cause of death among children younger than 5 years (1).

The burden and mortality of respiratory infections are unevenly distributed across geographic and socioeconomic lines. This pattern is largely driven by differences in access to healthcare services, vaccination coverage, nutritional status, and exposure to environmental risk factors such as household air pollution and ambient particulate matter (15, 16). In 2023, the highest pneumonia mortality rates among children younger than 5 years were reported in sub-Saharan Africa, reflecting the combined effects of limited healthcare infrastructure, higher prevalence of comorbidities, and delayed access to medical care (5).

RSV, HMPV, influenza, parainfluenza, rhinovirus, and adenovirus—the most detected pediatric LRTIs—account for a substantial proportion of hospital admissions for bronchiolitis and pneumonia in infants and young children, especially during seasonal epidemics (2, 17). Most respiratory viruses exhibit marked seasonal patterns, though the timing and intensity of circulation vary across geographic regions and climatic zones (18). RSV infections typically peak during the winter months (December-March in the Northern Hemisphere; April-September in the Southern Hemisphere) (19). Influenza viruses similarly show a winter peak in temperate regions and year-round circulation in tropical zones (20). Parainfluenza viruses exhibit seasonal patterns that vary by viral subtype and geographic region. For example, Parainfluenza virus type 3 typically peaks between spring and early summer in the United States (21), between April and May in Scotland (22), during autumn in Korea (23), and from late winter to spring in South Africa (24). Parainfluenza virus type 4 has been reported to circulate mainly between autumn and winter in the United States (21) and from late summer to early winter in South Africa (24).

Understanding regional differences in viral seasonality is essential for anticipating epidemic peaks, optimizing healthcare resource allocation, and guiding preventive strategies, including vaccination campaigns and passive immunization programs. This epidemiological knowledge is particularly relevant for timing the administration of long-acting monoclonal antibodies and vaccines in early childhood.

### 2.2 Risk groups in pediatrics

Underlying medical conditions are pivotal in determining the risk of severe disease and hospitalization among infants and children with viral respiratory infections. Prematurity is the most commonly reported comorbidity among pediatric patients hospitalized for LRTI. Additional risk factors include low birth weight, maternal smoking during pregnancy or in the postnatal period, exposure to secondhand smoke, indoor air pollution, bronchopulmonary dysplasia (BPD), congenital heart disease, immunodeficiency, and neuromuscular disorders (25, 26).

Prematurity is a well-recognized risk factor for severe viral respiratory disease due to the immaturity of the pulmonary and immunological systems. Preterm infants exhibit incomplete lung development, reduced airway caliber, impaired mucociliary clearance, and an immature immune response, all of which increase susceptibility to LRTIs and more severe clinical presentations (27). BPD, a chronic lung disease of prematurity characterized by arrested alveolar development and structural abnormalities of the lung parenchyma, further increases susceptibility to viral respiratory infections.

Children with BPD often have reduced pulmonary reserve and greater airway reactivity, which may lead to more severe disease courses and increased healthcare utilization during respiratory infections.

Evidence from large hospitalization studies shows that BPD is associated with longer hospital stays and a higher risk of in-hospital complications and mortality among infants hospitalized for viral respiratory infections (27). Children with congenital heart disease (CHD) are at increased risk of complications and mortality following viral respiratory infections. Several mechanisms contribute to this vulnerability, including compromised cardiopulmonary function, altered pulmonary circulation, ventilation-perfusion mismatch, pulmonary hypertension, and, in some cases, nutritional deficiencies that may impair immune responses (28, 29). Observational studies demonstrate that CHD significantly increases the risk of severe outcomes. These outcomes include prolonged hospitalization, intensive care admission, and death among children with viral respiratory infections (27).

Epidemiological analyses of hospitalized infants younger than 2 years have shown that viral respiratory infections are among the leading causes of pediatric hospitalization. In a large cohort study of more than 66,000 hospitalizations, approximately 10% of children had relevant underlying conditions, including prematurity, BPD, or congenital heart disease. In this population, BPD and congenital heart disease were among the most important predictors of adverse outcomes, including longer hospital stays, in-hospital complications, and mortality. Additionally, pneumonia and the need for invasive medical procedures were major determinants of increased morbidity and mortality in these patients (27).

A recent systematic review and meta-analysis further evaluated risk factors for poor outcomes, defined as the need for supplemental oxygen, mechanical ventilation, intensive care unit admission, prolonged hospitalization, or mortality, among children younger than 5 years hospitalized with acute LRTIs caused by RSV, influenza, and SARS-CoV-2 (30). Among RSV-associated LRTIs, the strongest predictors of severe outcomes were neurological disease, Down syndrome, chronic lung disease, immunocompromised status, prematurity, and congenital heart disease. Additional risk factors included age younger than 2 months, age younger than 6 months, viral coinfection, low birth weight, and underweight status. For influenza-associated LRTIs, age between 6 and 24 months and the presence of chronic medical conditions were major risk factors for severe outcomes. For SARS-CoV-2-associated LRTIs, cardiovascular disease, immunosuppression, chronic kidney disease, diabetes, and hypertension were significant predictors of mortality (30). Identifying vulnerable populations at risk of severe viral respiratory infections is essential for informing the implementation of preventive strategies, including vaccination, passive immunization with monoclonal antibodies, and targeted public health interventions. These risk factors can help prioritize access to high-cost interventions when universal strategies are not yet feasible. However, although prematurity and chronic medical conditions significantly increase the risk of severe RSV disease, the majority of RSV-related hospitalizations occur in previously healthy term infants without identifiable risk factors at birth (25). This epidemiological observation has important implications for prevention policies and support the transition for risk-based approaches toward universal population-based preventive strategies.

### 2.3 Long-term respiratory outcomes

The potential impact of early-life viral respiratory infections on long-term respiratory health has been extensively studied over the past decades. Observational and longitudinal cohort studies have shown that viral LRTIs in infancy are associated with an increased risk of recurrent wheezing and asthma later in childhood (31–34). Among respiratory pathogens, RSV and rhinovirus are the most consistently implicated in subsequent wheezing disorders (35, 36).

RSV is among the viral pathogens most frequently associated with early-life wheezing and bronchiolitis (36). Epidemiological studies have shown that infants with RSV-related LRTIs have a 25%–80% higher risk of developing recurrent wheezing or asthma than uninfected controls, and these effects persist for several years after the acute infection (37–39). In a population-based cohort study, Stein et al. found that children who experienced RSV-related LRTIs in infancy were 3.2 times more likely to develop infrequent wheezing and 4.3 times more likely to develop frequent wheezing by age 6 years (37). The strength of this association declined with age and was no longer statistically significant by adolescence (37).

Early studies also showed that severe RSV infection during the first year of life is associated with a higher incidence of wheezing in the first 4 years of life (40). Experimental and mechanistic studies have provided further insight into the biological pathways linking RSV infection to long-term airway dysfunction. Three-dimensional human lung organoid models have demonstrated that prenatal or early-life RSV infection can alter fetal lung development, reducing the proportion of ciliated epithelial cells, 185 increasing reactive club cell—also known as bronchiolar exocrine cells-proliferation, and disrupting the actin cytoskeleton (41). RSV infection may also alter the expression of key airway receptors, including increased phosphorylation of β2-adrenergic receptors (β2AR) and transient receptor potential vanilloid 1 (TRPV1) in epithelial and mesenchymal compartments (41, 42). These alterations may promote airway inflammation, hyperresponsiveness, and increased susceptibility to recurrent wheezing. Additional mechanisms contributing to airway hyperreactivity include the development of a pro-contractile phenotype characterized by elevated intracellular calcium concentrations in airway smooth muscle cells, a process implicated in both viral wheezing (43) and asthma pathogenesis (44). Indeed, increased TRPV1 expression and higher intracellular Ca2+ levels have been observed in the bronchial epithelial cells of asthmatic children compared with age-matched healthy controls (44).

Rhinovirus infection has also been strongly associated with the development of recurrent wheezing and asthma in childhood. In a study by Rakes et al., among children presenting to the emergency department with acute wheezing, rhinovirus was detected in 41% of infants younger than 2 years and in 71% of children older than 2 years with a history of wheezing (45). Longitudinal cohort studies have further demonstrated that Rhinovirus infections during the first 3 years of life are associated with an early 10-fold increase in the risk of developing asthma by age 6 (46). The mechanisms by which Rhinovirus infection contributes to asthma development are not fully understood but appear to involve virus-induced airway inflammation and immune dysregulation. Rhinovirus infections can promote a T-helper 2 (Th2) airway inflammatory response, characterized by the production of cytokines that drive allergic airway inflammation (47). Furthermore, infection stimulates airway epithelial cells to release thymic stromal lymphopoietin (TSLP), an epithelium-derived cytokine that promotes the differentiation of naïve T cells into Th2 lymphocytes (48, 49). This immune polarization may facilitate the development of airway hyperresponsiveness and allergic inflammation.

Evidence on the long-term respiratory consequences of HMPV infection is limited. However, emerging studies suggest that early-life LRTIs caused by this virus may also be associated with an increased risk of recurrent wheezing and asthma. In a cohort study, Coverstone et al. reported a higher incidence of recurrent wheezing and asthma among children who experienced HMPV-associated LRTIs (50).

Furthermore, follow-up studies have suggested that early HMPV infection may predispose to bronchospasm at school age (51). Experimental evidence indicates that this association may be mediated by alterations in Th2 immune responses and impaired viral clearance after infection (52).

Overall, growing evidence suggests that severe viral respiratory infections in early life influence long term respiratory health through complex interactions among viral pathogenic mechanisms, host immune responses, and genetic susceptibility. Long-term respiratory sequelae may be more strongly associated with the timing of severe viral infection during critical periods of lung and immune development rather than with a specific viral pathogen. Understanding these interactions is particularly important given emerging preventive strategies that may modify not only acute disease but also long-term respiratory outcomes. However, definitive evidence is lacking to support the effectiveness of current preventive strategies (such as monoclonal antibodies or maternal vaccination) in mitigating the long-term risk of asthma. Thus, extended follow-up of exposed cohorts is needed.

## 3 Respiratory syncytial virus as a model pathogen

### 3.1 Virology and pathogenesis

RSV is an enveloped RNA virus in the Paramyxoviridae family, with a non-segmented, single- stranded, negative-sense genome (53). The viral genome encodes 11 proteins from 10 genes, including three surface glycoproteins [attachment glycoprotein (G), fusion (F) protein, and small hydrophobic (SH) protein], three nucleocapsid proteins [large (L) polymerase, N protein, and phosphoprotein (P)], two non-structural proteins (NS1 and NS2), transcription factors (M2-1 and M2-2), and a matrix (M) protein (54, 55). Among these, the G and F glycoproteins are key to viral entry, mediating attachment to and fusion with host epithelial cells. The F protein is highly conserved and is the primary target for neutralizing antibodies and current immunoprophylactic strategies. Circulating RSV strains are classified into two antigenic subgroups, RSV-A and RSV-B. These subgroups mainly differ in their G protein sequences and may exhibit variations in epidemiological patterns and disease severity (56).

RSV infection typically begins in the nasal epithelium, where infected epithelial cells release pro- inflammatory mediators and recruit innate immune cells, including monocytes, macrophages, and dendritic cells (DCs) (57). These cells produce a variety of cytokines, including tumor necrosis factor (TNF)-α, interleukin (IL)-1, IL-6, IL-8, IL-10, and IL-18, which contribute to airway inflammation and immune activation (57). Viral recognition is mediated by pattern recognition receptors, particularly Toll-like receptors (TLR2, TLR3, TLR4, TLR7, and TLR8), which trigger signaling pathways that regulate antiviral responses and the development of adaptive immunity (2).

Type I interferons (IFNs) are central to antiviral defense. However, RSV has evolved mechanisms to evade this response. The viral non-structural proteins NS1 and NS2 inhibit IFN signaling by forming a degradative complex, the NS degradosome, which targets host innate immune signaling proteins for degradation (58). In addition, the G and F proteins may impair IFN production, facilitate viral replication, and promote mucus hypersecretion (2).

The host's adaptive immune response develops approximately 4–7 days after infection and involves coordinated activation of T and B lymphocytes (57). DCs present viral antigens to T cells in regional lymph nodes, leading to activation of RSV-specific CD4+ Th cells and cytotoxic CD8+ T cells, and stimulating B cells to produce virus-specific antibodies (59). Neutralizing antibodies against the F protein are particularly effective at preventing viral entry, and the F protein is the main target of current monoclonal antibody and vaccine strategies (60). An imbalance between Th1 and Th2 immune responses have been associated with more severe disease, particularly in young children, where excessive Th2 polarization may promote airway inflammation, mucus production, and eosinophilic responses (61). The high conservation of the prefusion F protein and the central role of neutralizing antibodies targeting this epitope have enabled the development of long-acting monoclonal antibodies and pre F-based vaccines, positioning RSV as a model for structure-guided prevention of viral respiratory diseases.

### 3.2 RSV-driven innovation in preventive strategies

RSV is the leading cause of hospitalization and death from respiratory infections in the first year of life, representing a major global pediatric health burden (62). RSV-associated bronchiolitis and pneumonia are also linked to higher healthcare utilization and hospitalization costs than severe respiratory infections caused by other pathogens (63). Despite this substantial medical and economic impact, therapeutic options for RSV infection in infants remain limited. Current management of RSV-associated LRTIs is largely supportive, including maintaining adequate hydration and providing respiratory support as needed (64).

Currently, ribavirin is the only antiviral drug approved by the U.S. Food and Drug Administration (FDA) for treating severe RSV infection in hospitalized infants and young children (65). It is a synthetic nucleoside analog that inhibits viral replication by interfering with RNA polymerase activity; hence, it has a virustatic effect *in vitro* but no virucidal activity. Ideally, it should be administered as a continuous aerosol for 12–18 h per day for 3–7 days using a special small-particle aerosol generator (SPAG) to optimize delivery to the lower respiratory tract, starting within 24–48 h of the onset of illness (66, 67). However, in real-world settings, the clinical manifestations of severe viral bronchiolitis typically appear several days after infection begins, and at that point, a virustatic drug like ribavirin cannot affect viral replication. Indeed, there is extensive evidence that the viral load in the respiratory tract of immunocompetent infants declines rapidly, yet the disease process persists, primarily driven by the host's cytokine storm in response to the infection, which persists even after most of the virus has been cleared. For this reason, it is unlikely that ribavirin will significantly improve outcomes in most infants with RSV-related LRTIs (68). These concerns about limited efficacy, combined with high costs and potential toxicity (including teratogenic and embryogenic effects demonstrated in animal studies), have led to a revision of the original guidelines and restricted ribavirin use to immunocompromised patients whose immune systems are compromised and cannot clear the virus for longer periods of time (69–71).

In recent years, several antiviral compounds targeting different steps of the RSV replication cycle have been investigated. Entry inhibitors, which block the F protein and prevent viral membrane fusion with host cells, constitute the largest class of small-molecule antivirals in development. Several candidates, including enzaplatovir, presatovir, rilematovir, sisunatovir, and MDT-637, have shown antiviral activity but were discontinued in phase II or III clinical trials due to limited efficacy or safety concerns (72). Ziresovir, another fusion inhibitor, has demonstrated promising results in infants aged 1-month hospitalized with RSV infection, significantly reducing viral load and improving clinical symptoms compared with placebo (73). However, resistance mutations remain a challenge for this class of compounds (72).

Other antiviral strategies include polymerase inhibitors that target the viral RNA-dependent RNA polymerase complex responsible for genome replication and transcription. By interfering with viral RNA synthesis, these agents suppress viral replication after cell entry (72). Ribavirin was the first example of this class, but several newer polymerase inhibitors with improved potency and safety profiles are currently under investigation (74, 75). In addition, nucleoprotein inhibitors have emerged as a novel therapeutic approach. These agents interfere with the RSV nucleoprotein (N), which is essential for viral genome encapsidation and for formation of the replication complex. By disrupting nucleocapsid assembly or stability, these molecules impair viral replication and reduce viral propagation (76). Because the nucleoprotein is highly conserved across RSV strains, inhibitors targeting this protein provide a higher genetic barrier to resistance than entry inhibitors (72). Although most RSV antivirals are still in clinical development, they may complement preventive strategies in the future, particularly for treating severe infections in high-risk pediatric populations.

Developing effective RSV vaccines has long been challenging. In the 1960s, a formalin-inactivated RSV vaccine with alum was tested in infants and young children. However, vaccinated children who were later exposed to natural RSV infections developed more severe disease, including higher rates of hospitalization and pneumonia, than unvaccinated children (77–79). This phenomenon, known as enhanced respiratory disease (ERD), was later attributed to poorly neutralizing antibodies and a predominant Th2 immune response (80–82). ERD led to a prolonged pause in RSV vaccine development, particularly for pediatric populations (83). Over the past decade, advances in understanding RSV molecular structure and immune responses, particularly the identification of the prefusion conformation of the F protein, have renewed interest in preventive strategies (53). These discoveries, together with the persistent global burden of RSV in infants and young children, have driven the development of novel vaccines and monoclonal antibodies aimed at preventing RSV infection and reducing its associated morbidity and mortality.

## 4 Lessons learned from RSV prevention strategies

### 4.1 Passive immunization: first-generation monoclonal antibodies

Hyperimmune immunoglobulins and monoclonal antibodies have shown promising results as preventive strategies against RSV infection in vulnerable pediatric populations. In the early 1990s, two randomized controlled trials in high-risk infants demonstrated that monthly administration of RSV intravenous immune globulin (RSV-IGIV) during the RSV season reduced hospitalization rates by 40%–65% (84, 85). However, subsequent studies reported an increased risk of adverse outcomes, including higher morbidity and mortality, among infants with congenital heart disease receiving RSV-IVIG (86).

Palivizumab (AstraZeneca AB; Sweden) is a humanized monoclonal immunoglobulin G1 (IgG1) antibody produced by recombinant DNA technology that targets the F protein (87). Introduced in 1998, palivizumab was the first effective monoclonal-based strategy to prevent severe RSV disease in high-risk pediatric populations. By binding to the F protein, the antibody inhibits viral fusion with host cell membranes and blocks viral entry into respiratory epithelial cells (87).

Palivizumab prophylaxis was primarily recommended for infants at increased risk of severe RSV infection, including premature infants, children with bronchopulmonary dysplasia, and those with hemodynamically significant congenital heart disease. In select cases, it may also be considered for children with other high-risk conditions, such as cystic fibrosis, Down syndrome, congenital diaphragmatic hernia, neuromuscular disorders, or immunodeficiency (88, 89). However, due to its high cost and the need for multiple doses, palivizumab prophylaxis is limited to a small proportion of the pediatric population, covering only about 4%–6% of children at risk of severe RSV disease (90). Monthly intramuscular injections of palivizumab during the RSV season have been shown to reduce RSV-related hospitalizations by about 50% compared with placebo (90). Beyond its effect on acute disease, several studies have examined whether RSV prophylaxis may influence long-term respiratory outcomes. Randomized controlled trials in preterm infants have suggested that palivizumab prophylaxis reduces the incidence of recurrent wheezing in the first years of life (91, 92). Additional studies have reported that prophylaxis could decrease the risk of recurrent wheezing by up to 80% in non-atopic children, although this protective effect was not observed in infants with a family history of atopy (93). Longer-term follow-up studies have yielded mixed results. A multicenter prospective study of preterm infants born at 33–35 weeks’ gestation found that palivizumab prophylaxis significantly reduced the incidence of recurrent wheezing up to age 6 but did not prevent the development of atopic asthma (94).

More recent data from a prospective multicenter case-control study suggested that palivizumab prophylaxis does not significantly influence the risk of recurrent wheezing or asthma at age 10 (95).

One possible explanation is that the impact of RSV infection on airway disease is most pronounced in early childhood, particularly during the first six years of life (96). Furthermore, in the presence of atopic predisposition, the pathogenetic role of the infection may be overshadowed by activation of high-T2 pathways, especially when these pathways reach full maturity, i.e., after the preschool years. Although palivizumab was a major milestone in RSV prevention, its limited population coverage, need for repeated dosing, and high costs have underscored the need for broader, more durable preventive strategies. These limitations have spurred the development of next-generation monoclonal antibodies with longer half-lives and broader population coverage. Although palivizumab represented a major advance in RSV prevention, recent American Academy of Pediatrics (AAP) recommendations did no longer routinely recommend for use from December 31, 2025 (88), while WHO guidance now instead recommends the use of a long-acting RSV prevention mAb such as those discussed below (97).

### 4.2 Next-generation monoclonal antibodies

Advances in antibody engineering have enabled the development of next-generation monoclonal antibodies with extended half-life, allowing seasonal protection with a single administration. These innovations mark a major step forward compared with first-generation agents such as palivizumab. 320 Nirsevimab (Sanofi Winthrop Industrie; France) is a recombinant human IgG1 monoclonal antibody that targets a highly conserved epitope known as antigenic “site zero” (ø) on the prefusion RSV F protein (6). Structural modifications to the Fc region prolong its half-life, enabling protection for approximately 5 months and covering an entire RSV season with a single intramuscular dose (6). It is approved by both the European Medicines Agency (EMA, October 2022) (98) and the U.S. Food and Drug Administration (FDA, July 2023) (99) for use in all infants entering their first RSV season, regardless of gestational age or underlying conditions (100, 101). It is also approved in an increasing number of mostly, but not only, high-income countries (102). In addition, it is recommended for select high-risk children up to 24 months of age entering their second season (83, 103, 104). Clinical trials have demonstrated high efficacy, with reductions of 70%–80% in RSV-associated LRTIs and hospitalizations (105). Real-world studies have further confirmed these findings, reporting effectiveness of 80%–90% in preventing severe RSV disease and hospital admissions across different healthcare settings (105). Safety profiles have been favorable, with no significant increase in adverse events compared to placebo (106). Although long-term data are still limited, emerging evidence suggests a potential reduction in post-bronchiolitis wheezing, supporting the hypothesis that early RSV prevention may influence subsequent respiratory outcomes (107, 108).

Clesrovimab (Merck; USA) is another long-acting RSV-neutralizing monoclonal antibody that targets antigenic site IV of the F protein and is active in both prefusion and postfusion conformations (7). It has a prolonged half-life (approximately 2–3 months) and is administered as a single fixed-dose intramuscular injection regardless of body weight (8). Recently approved by the FDA (June 2025), it offers an alternative prevention strategy for infants during their first RSV season (109). It has also received a Positive Opinion from the EMA (110). In phase 2b/3 trials, clesrovimab demonstrated approximately 60% efficacy in preventing medically attended RSV-associated LRTIs and over 90% efficacy against RSV-related hospitalizations (111). Its safety profile was comparable to placebo (111). A phase 3, randomized, palivizumab-controlled trial (SMART) 342 showed that a single dose of clesrovimab had an efficacy and safety profile like monthly palivizumab in pediatric high-risk populations (112). Although clinical trial results are encouraging, real-world effectiveness data for clesrovimab remain limited because its recent introduction into clinical practice (111, 112).

Other candidates, including motavizumab and suptavumab, were discontinued due to limited efficacy or safety concerns observed during clinical development (113). These failures have underscored the importance of targeting conserved epitopes on the prefusion F protein and ensuring a high barrier to resistance. The discontinuation of suptavumab provides a clear example of how spontaneous viral mutations may compromise monoclonal antibody effectiveness. Despite targeting a theoretically conserved epitope on the RSV F protein, naturally occurring mutations within circulating RSV-B strains reduced susceptibility and led to clinical failure (114). The RSV F protein is highly conserved between RSV A and B subtypes and exhibits substantial genetic and antigenic stability. However, a potential risk remains for the emergence of antibody-resistant escape mutations among circulating RSV strains (114). Continuous molecular surveillance is therefore essential to enable early identification of escape variants, monitor changes in epitope conservation, and preserve the effectiveness of current immunoprophylactic strategies. Next-generation monoclonal antibodies have transformed RSV prevention by shifting from a risk-based to a population-based approach. Their single-dose regimen, broad eligibility, and high effectiveness make them a cornerstone of current pediatric RSV prevention strategies and a model for developing immunoprophylaxis against other respiratory viruses. However, specific challenges, including manufacturing capacity, costs, and integration into existing immunization schedules, must be addressed to fully realize their global impact, particularly in low- and middle-income countries.

### 4.3 Active immunization strategies

After the failure of the formalin-inactivated RSV vaccine, early active immunization efforts focused on purified F-protein subunit vaccines. However, these formulations showed insufficient immunogenicity in key target populations, including young infants and pregnant women, limiting their clinical applicability (115–119). Live-attenuated and intranasal RSV vaccines have also been extensively investigated, particularly for pediatric use, because they can induce mucosal immunity. However, their development has been challenged by the need to balance adequate immunogenicity with acceptable safety and reactogenicity in infants, as well as by interference from maternal antibodies and concerns about genetic stability and transmissibility (120–124).

Recent advances in structural vaccinology, particularly the identification of the prefusion conformation of the F protein, have revitalized vaccine development, yielding multiple novel approaches. These include recombinant and chimeric live-attenuated vaccines, preF protein-based subunit vaccines, viral vector vaccines, and mRNA-based approaches (123–125). Phase I and II clinical trials have shown that mRNA-based vaccines (e.g., mRNA-1345) and live-attenuated intranasal candidates (e.g., RSV/ΔNS2/Δ1313/I1314L) are generally safe and immunogenic in pediatric populations, both RSV-seropositive and seronegative (126–128). In a phase 1 trial involving RSV-seropositive children aged 12–59 months, the investigational preF mRNA vaccine mRNA-1345 demonstrated an acceptable safety profile and induced robust neutralizing antibody responses against both RSV-A and RSV-B (127). Vaccination preferentially boosted antibodies directed against the prefusion F conformation, the principal target of protective humoral immunity, supporting the ability of mRNA platforms to precisely focus immune responses toward highly conserved neutralizing epitopes (127). These findings highlight the potential of mRNA technologies to accelerate the development of next-generation pediatric RSV vaccines. Some live-attenuated intranasal candidates (e.g., RSV/ΔNS2/Δ1313/I1314L) have progressed to phase III trials in infants, although current evidence remains uncertain regarding their impact on clinically relevant outcomes, such as medically attended LRTIs (118, 126, 128). Additional live-attenuated or chimeric vaccines (e.g., MV-012-968 and BLB-201) and viral vector vaccines (e.g., Ad26.RSV.preF) have also demonstrated promising safety and immunogenicity profiles in early clinical trials in pediatric populations (129–132).

The development of maternal immunization strategies has been a major breakthrough in RSV prevention. The bivalent preF vaccine Abrysvo (RSVpreF; Pfizer) is the first RSV vaccine approved for administration during pregnancy to protect infants through passive transplacental antibody transfer (133). In the phase 3 MATISSE trial, maternal vaccination between 24 and 36 weeks of gestation demonstrated high efficacy against severe medically attended LRTIs, with protection of approximately 82% within the first 3 months of life and 70% within the first 6 months (133). Against less-severe medically attended LRTIs, the vaccine demonstrated an efficacy of 58% within the first three months of life and 49% in the first six months (134). Efficacy declines after six months for medically attended LRTIs across all severity levels. Abrysvo has shown a favorable safety profile, although initial analyses reported a small, non-significant increase in preterm births in the vaccinated group (134), which has not been confirmed in more recent post-marketing analyses. Indeed, subsequent studies and subgroup analyses have suggested that this result varies across geographic settings, with a potentially higher risk observed in low- and middle-income countries, particularly in sub-Saharan Africa (9). Modeling studies have supported the use of maternal vaccination in the third trimester to optimize both safety and effectiveness, especially in high-risk populations (135). The landscape of infant protection against RSV has fundamentally shifted with the introduction of safe and efficacious immunization technologies (136). Current global strategies lean on seasonal administration of the bivalent prefusion F maternal vaccine (RSVpreF) between 32 and 36 weeks of gestation (136). This specific timeframe is widely recommended by regulatory authorities to maximize passive transplacental antibody transfer and safeguard infants during their highly vulnerable first months of life (136). However, emerging clinical evidence introduces a compelling paradigm shift, suggesting that maternal vaccine effectiveness may be highly optimized when administered even earlier in pregnancy—specifically between 28 and 32 weeks of gestation—rather than waiting for the traditional late-pregnancy window. Maximizing the potential of this maternal vaccine, alongside infant immunoprophylaxis like nirsevimab, is critical to addressing the severe global burden of RSV-associated lower respiratory tract infections in young children (136).

This timing allows at least six weeks for efficient transplacental transfer of maternal antibodies and results in higher infant antibody concentrations at birth. Consistent with this observation, recent studies have demonstrated superior protection among infants whose mothers received vaccination earlier during the recommended gestational window (137). Therefore, while current recommendations allow administration later in pregnancy (10), vaccination between 28 and 32 weeks may maximize infant protection against RSV during the first months of life.

Active immunization strategies against RSV are rapidly evolving, with multiple platforms in development. Alongside long-acting monoclonal antibodies, maternal vaccination is a cornerstone of modern RSV prevention and a model for protecting vulnerable pediatric populations against viral respiratory infections. In addition to lower cost, easier storage and transportation, and activation of mucosal immunity, another strategic advantage of vaccine platforms is the rapid development of multivalent constructs that can target multiple respiratory viruses (e.g., RSV, influenza, HMPV, SARS-CoV-2) with a single administration. These products are likely to enter the market in the near future, and future research will be needed to clarify how best to use maternal vaccines and infant monoclonal antibodies together by evaluating local epidemiology, health system capacity, and cost-effectiveness.

## 5 Translating RSV lessons to other respiratory viruses

### 5.1 Influenza

Influenza viruses are major respiratory pathogens in the Orthomyxoviridae family, primarily represented by influenza A and B viruses. These are single-stranded, negative-sense RNA viruses with a segmented genome of eight RNA segments that encode key structural and non-structural proteins, including hemagglutinin (HA), neuraminidase (NA), nucleoprotein, and polymerase complexes (11, 138). HA mediates viral entry into host cells, whereas NA facilitates the release of newly formed virions, making both proteins central targets for vaccines and antiviral therapies (138). In common with 428 RSV there are two non-structural proteins (NS1 and NS2) (138).

Influenza viruses cause seasonal epidemics and occasional pandemics, contributing substantially to global morbidity and mortality (139). In temperate regions, influenza activity typically peaks in winter, whereas in tropical areas, transmission may occur year-round (12). Disease burden is particularly high among children younger than 5 years and among those with underlying medical conditions, in whom complications such as pneumonia significantly increase the risk of hospitalization and death (12, 140). Children also play a key role in viral transmission within households and communities, amplifying the spread of infection (141, 142).

A major challenge in influenza prevention is the virus's high mutation rate, driven by antigenic drift and shift, which continuously generates new strains and alters population immunity (143–145). As a result, influenza vaccines require annual reformulation based on global surveillance data coordinated by the World Health Organization (WHO) (146). Vaccination remains the cornerstone of prevention, with routine immunization recommended starting at 6 months of age, particularly for high-risk children and their contacts (147, 148). Despite widespread use, vaccine effectiveness varies annually, typically ranging from 40% to 60%, depending on the match between the vaccine and circulating strains (149).

Recent surveillance data indicate variable effectiveness across seasons, although vaccination consistently reduces disease severity and healthcare burden (150).

Lessons learned from RSV underscore the importance of targeting conserved viral epitopes and developing long-acting immunoprophylaxis strategies. In this context, monoclonal antibodies are emerging as promising tools for influenza prevention and treatment. Several neutralizing monoclonal antibodies targeting HA, NA, and the ion channel protein M2 are under investigation (150). Anti-HA monoclonal antibodies (e.g., CR6261, FI6, and MEDI8852) block viral entry and show broad neutralizing activity across multiple influenza A subtypes (151–153). Anti-NA monoclonal antibodies (e.g., NC10 and flunavirumab) inhibit viral release and limit spread by preventing cleavage of sialic acid residues (153). These antibodies have shown broad-spectrum activity against influenza A and B viruses, particularly against drug-resistant variants (154). Anti-M2 monoclonal antibodies block viral replication and prevent the release of viral RNA into the host cell (155). These antibodies offer potential for broader-spectrum activity by targeting a highly conserved protein (156).

As with early RSV prevention, several pressing challenges remain, including the potential for resistance and variable clinical efficacy. Furthermore, data on pediatric populations are limited because most studies to date have been conducted in immunocompromised adults (156, 157). Nonetheless, the development of broadly neutralizing monoclonal antibodies targeting conserved viral structures may help overcome the limitations of strain-specific vaccines. While influenza vaccination remains essential, its strain-specific nature and variable effectiveness underscore the need for complementary strategies. In this context, broadly neutralizing monoclonal antibodies and universal vaccine platforms represent promising tools to overcome antigenic variability and enhance protection, especially in vulnerable pediatric populations. By applying the RSV model, such as focusing on conserved epitopes and exploring long-acting formulations, future influenza prevention strategies may become more durable and longer-lasting.

### 4.2 SARS-CoV-2

Since 2020, coronavirus disease 2019 (COVID-19) has caused substantial morbidity and mortality worldwide, including approximately 38,000 hospitalizations among children and adolescents during September 2023–August 2024 (158). SARS-CoV-2, a member of the Coronaviridae family, is an enveloped, positive-sense single-stranded RNA virus that primarily targets the respiratory tract but can also affect multiple organs and systems. The viral replication cycle begins when the S1 subunit of the spike (S) glycoprotein binds to the angiotensin-converting enzyme 2 (ACE2) receptor, facilitating viral entry into host cells via membrane fusion or endocytosis (159). Subsequent viral replication and host immune responses contribute to clinical manifestations ranging from asymptomatic infection to severe disease, including acute respiratory distress syndrome and multisystem inflammatory syndrome in children (MIS-C) (160).

Current AAP statements recommend COVID-19 vaccination for all children ages 6 through 23 months who are at high risk of severe disease (161), and WHO guidance similarly recommends vaccination for children with significant comorbidities or severe obesity (162). High-risk conditions include chronic pulmonary or cardiovascular disease, gastrointestinal disorders, hepatic or hematologic disease, metabolic disorders, obesity, neurologic or neurodevelopmental, disorders, immunosuppressive conditions, and rheumatologic or autoimmune disease (161). In addition, broader pediatric vaccination strategies have been implemented in many countries to reduce transmission, prevent severe outcomes, and limit the emergence of variants of concern (163).

Monoclonal antibodies targeting key SARS-CoV-2 proteins have been used to treat COVID-19 in individuals at risk of progression to severe disease, although pediatric data remain limited.

Bamlanivimab (AbCellera Biologics and Eli Lilly, USA) was the first monoclonal antibody to receive emergency use authorization in November 2020, followed by the combination of bamlanivimab and etesevimab (164). Both agents bind to the receptor-binding domain (RBD) of the spike protein, thereby inhibiting viral attachment and entry into host cells (165). These therapies have been authorized for adolescents aged 12 years and older and weighing more than 40 kg (166). However, in June 2021, the US Office of the Assistant Secretary for Preparedness and Response (ASPR) paused the distribution of bamlanivimab and etesevimab together and of etesevimab alone (to pair with the existing bamlanivimab supply) due to the increase in circulating variants.

Despite a European case series published in 2023 that evaluated monoclonal antibody use in children with a median age of 5.4 years (range 0–13 years) and reported a favorable safety profile (167), these treatments are not currently authorized for pediatric patients under 12 years of age (168). Furthermore, the clinical utility of several monoclonal antibodies has been significantly reduced by the emergence of viral variants with reduced susceptibility. The FDA revoked authorization for bamlanivimab monotherapy due to the high prevalence of resistant SARS-CoV-2 strains (169). Similarly, sotrovimab, another monoclonal antibody previously authorized for individuals older than 12 years, had its emergency use authorization withdrawn in 2022 because of the spread of resistant variants (170).

Nevertheless, emerging evidence continues to support the potential role of monoclonal antibodies in select pediatric populations. A recent study of high-risk children aged 6–12 years with mild-to-moderate COVID-19 demonstrated the safety and tolerability of a single sotrovimab dose (170).

Ongoing research focuses on developing next-generation monoclonal antibodies with broader neutralizing activity against evolving variants and on optimizing their role alongside vaccination strategies for vulnerable pediatric groups.

### 4.3 Emerging targets

Rhinoviruses are the most prevalent respiratory pathogens worldwide, causing upper and lower respiratory tract infections across all age groups (171). In children, early-life rhinovirus infections are strongly linked to long-term respiratory outcomes, including recurrent wheezing and asthma, and contribute significantly to hospitalizations and chronic respiratory morbidity (46, 172). These viruses circulate year-round, with seasonal peaks in autumn and spring in temperate regions and less pronounced seasonality in tropical climates (173, 174). Despite their substantial burden, no licensed vaccines are available (175). Vaccine development is hindered by extensive antigenic diversity across the three rhinovirus species (A, B, and C), which differ in capsid structure and receptor usage (176, 177).

Early formalin-inactivated vaccines were immunogenic, inducing both systemic and mucosal responses, but provided only type-specific protection (178). More recent preclinical studies have shown that highly multivalent inactivated vaccines, including up to 50 serotypes, can elicit broad immune responses, supporting the feasibility of this approach (179). Subunit vaccines based on capsid proteins have also demonstrated type-specific immunogenicity, although their effectiveness is limited by antigenic variability (175, 180). Future strategies include live-attenuated vaccines, virus-like particles, and hybrid platforms designed to enhance immunogenicity while maintaining safety (175).

Alternative approaches aim to block viral entry by targeting host receptors such as ICAM-1; however, monoclonal antibodies against these receptors have shown limited *in vivo* efficacy to date (181, 182).

As discussed above, HMPV is another important pediatric respiratory pathogen, responsible for more than 14 million LRTIs annually in children under 5 years of age (183, 184). This virus is classified into two major genetic lineages (A and B) that share substantial protein homology, enabling identification of conserved targets for vaccine development (185, 186). Traditional vaccine approaches have faced significant challenges. Live-attenuated vaccines have shown insufficient immunogenicity, while inactivated vaccines raise concerns about ERD, as seen with the early RSV vaccine (187, 188).

Recombinant protein and virus-like particle vaccines targeting the fusion (F) protein have demonstrated strong neutralizing antibody responses in animal models, although the duration of protection remains limited (189). Novel strategies, including viral vector-based and lipid nanoparticle platforms, are under investigation (13). Monoclonal antibodies targeting the HMPV F protein have shown promising prophylactic and therapeutic effects in preclinical models (190, 191). Notably, cross-neutralizing antibodies, such as 54G10 and MPE8, can provide protection against both RSV and HMPV, highlighting the potential of targeting conserved epitopes across related viruses (192, 193). MPV364 demonstrated potent neutralizing activity against both HMPV A and B genotypes, with higher *in vitro* activity than several previously described antibodies (192). Prophylactic administration of MPV364 significantly reduced pulmonary viral replication in murine models, supporting the feasibility of mAb-based prevention strategies for HMPV (192). Structural analyses further suggest that highly conserved antigenic site III epitopes within the HMPV F protein may represent particularly attractive targets for future vaccina and monoclonal antibody development (192).

Parainfluenza viruses are also major contributors to pediatric respiratory morbidity and mortality worldwide (194). Parainfluenza virus type 1 is a leading cause of croup, bronchiolitis, and pneumonia in young children, accounting for a substantial number of hospitalizations and deaths globally (195, 196). Despite this burden, no licensed vaccines are currently available. Early inactivated vaccines failed to confer protection, and live-attenuated candidates have shown limited immunogenicity 549 (197, 198). More recently, novel strategies have shown encouraging results. Intranasal vaccines based on Sendai virus vectors (murine parainfluenza type 1) have demonstrated good tolerability and immunogenicity in children (199). For parainfluenza virus type 3, both live-attenuated and subunit vaccines have demonstrated favorable safety profiles and immunogenicity in clinical and preclinical studies (200, 201). Recent structural and preclinical studies have identified the prefusion conformation of parainfluenza type 3 fusion (F) protein as a promising target for monoclonal antibody development (202). Among the most extensively candidates, PI3-E12 bins conserved prefusion-specific epitope and exhibits potent neutralizing activity against parainfluenza type 3 (202). In animal models, administration of PI3-E12 significantly reduced viral replication when given either prophylactically or shortly after infection, supporting the feasibility of antibody-based prevention and treatment strategies (202). These observations highlight the successful RSV paradigm, where stabilization and targeting of prefusion F protein revolutionized immunoprophylaxis, and suggest that structurally constrained fusion proteins may represent common targets across multiple respiratory viruses.

### 5.4 Common threads in the viral biology of respiratory pathogens

Despite belonging to diverse taxonomic families, the above-mentioned pathogens exhibit profound similarities in their structural biology, viral entry mechanisms, and strategies for host immune evasion. RSV, HMPV, and Parainfluenza viruses, enveloped RNA viruses, are members of the Mononegavirales order (families Pneumoviridae and Paramyxoviridae, respectively), sharing non segmented, single-stranded, negative-sense RNA genomes (53, 185, 195). In contrast, Influenza shares the negative sense RNA polarity but shows eight distinct single-stranded segments (139). Like human Rhinoviruses, which belong to the family Picornaviridae, SARS-CoV-2 (family Coronaviridae) is a positive-sense, single stranded RNA virus (160, 171). Conversely, the latter relies on a lipid envelope decorated with surface glycoproteins, whereas Rhinoviruses are non-enveloped, protein-capsid viruses (159, 171).

Despite these genomic differences, reliance on an RNA-dependent RNA polymerase complex across these pathogens result in high mutation rates, driving antigenic variability that complicates long-term vaccine efficacy. A critical biological convergence among these respiratory viruses is their reliance on class I viral fusion (F) proteins to mediate attachment and entry into host epithelial cells. RSV, HMPV, and Parainfluenza employ a highly analogous dual-protein surface system. They use an attachment glycoprotein (such as G in RSV or HA-NA in parainfluenza) to bind to host cells, alongside a dedicated F protein. The F proteins of RSV and HMPV, in particular, exhibit remarkable structural homology and share a highly transient, metastable “prefusion” conformation that exposes conserved neutralizing epitopes (such as antigenic “site zero”, ø) (203, 204).

Thanks to HA and spike (S) glycoprotein, respectively, Influenza and SARS-CoV-2, operate as single, multifunctional class I fusion machines. Like the paramyxovirus F protein, HA and S undergo massive structural rearrangements from a metastable prefusion state to a post-fusion state upon binding to host receptors, pulling the viral and host membranes together to form an entry pore (205). Because structural stability in the prefusion state preserves the most vulnerable, highly conserved epitopes, stabilizing this specific conformation can serve as a target for designing next-generation, neutralizing mAbs and universal vaccines. Importantly, not all conserved viral regions represent equally suitable therapeutic targets. The success of RSV immunoprophylaxis suggests that optimal targets are not merely conserved but functionally constrained, meaning that significant mutations would compromise viral fitness (206). Identifying analogous constrained regions within influenza, SARS-CoV-2, and other respiratory viruses may be more relevant than simply searching for sequence conservation.

**Figure 1:**
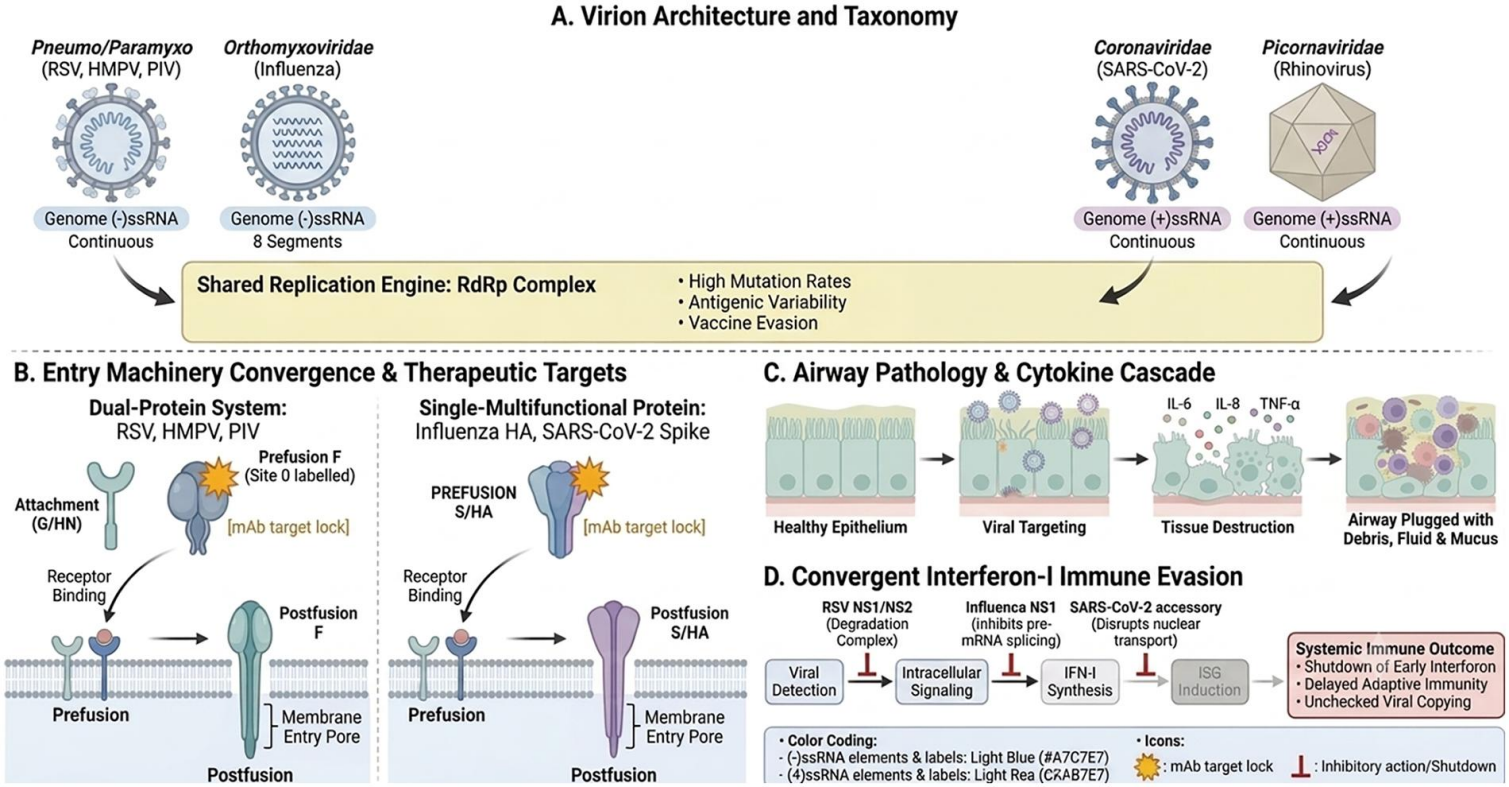
Shared biological archetypes and pathogenic convergence of respiratory viruses. Comparative schematic of the structural, replicative, and immunopathological mechanisms shared by major respiratory pathogens. **(a)** Virion Architecture and Taxonomy: Classification of (-)ssRNA (RSV, HMPV, PIV, Influenza) and (+)ssRNA (SARS-CoV-2, Rhinovirus) viruses. Despite taxonomic divergence, all share an RNA-dependent RNA Polymerase (RdRp) replication engine prone to high mutation rates, antigenic variability, and vaccine evasion. **(b)** Entry Machinery Convergence and Therapeutic Targets: Comparison of viral entry strategies, categorized into dual-protein attachment/fusion systems (e.g., RSV F protein) and single-multifunctional protein trimers (Influenza HA, SARS-CoV-2 Spike). Monoclonal antibody (mAb) target locks exploit vulnerable prefusion conformations prior to membrane entry pore formation. **(c)** Airway Pathology & Cytokine Cascade: Timeline of epithelial damage where viral targeting triggers a pro-inflammatory cytokine cascade (IL-6, IL-8, TNF-alpha), resulting in tissue destruction and airways plugged with debris, fluid, and mucus. **(d)** Convergent Interferon-I Immune Evasion: Distinct viral mechanisms yielding a shared systemic outcome. RSV NS1/NS2, Influenza NS1, and SARS-CoV-2 accessory proteins target different stages of the host immune response (signaling, IFN-I synthesis, and ISG induction), collectively causing early interferon shutdown, delayed adaptive immunity, and unchecked viral replication.

All these pathogens exhibit a primary tropism for the respiratory epithelium, initiating infection in the nasal mucosa and then moving to the lower respiratory tract. They selectively target ciliated epithelial cells, disrupting cellular architecture, downregulating mucociliary clearance, and inducing cellular apoptosis or syncytia formation. The epithelial damage triggers a cascade of pro-inflammatory cytokines and chemokines (such as IL-6, IL-8, and TNF-α) that can drive both direct viral replication and a hyper inflammatory host response, resulting in airway obstruction and plugging of cellular debris and mucus (2, 12, 158). Moreover, to successfully establish infection in the respiratory tract, these viruses have evolved mechanisms analogous to those that counteract the host's primary line of defense by the IFN-I pathway (2, 58). Thanks to NS1 and NS2, RSV forms a degradative complex that targets host innate signaling molecules (58). Influenza deploys NS1 to block host cellular pre-mRNA splicing and inhibit IFN synthesis (207). Similarly, SARS-CoV-2 encodes multiple accessory proteins (e.g., ORF6 and NSP1) that disrupt host nuclear transport, effectively blocking the expression of IFN-stimulated genes (208). Lastly, by shutting down IFN production during the early stages of infection, these pathogens delay adaptive immune activation, allowing unchecked initial replication and contributing to the immune dysregulation (209–211). Shared biological archetypes and pathogenic converge of the viruses are reported in Figure 1.

## 5 Conclusions

RSV has become a paradigm for the prevention of viral respiratory diseases in early childhood, illustrating both the challenges and opportunities of modern immunoprophylaxis. Historical issues, including vaccine-ERD associated with formalin-inactivated RSV vaccines, highlighted the importance of understanding viral pathogenesis and host immune responses. More recently, advances in structural vaccinology and antibody engineering have allowed the development of highly effective preventive strategies, including long-acting monoclonal antibodies and maternal prefusion F protein-based vaccines (53, 54, 77, 78, 83). The introduction of agents such as nirsevimab and clesrovimab, together with maternal RSV vaccination, has transformed prevention from a risk-based approach focused on selected vulnerable infants to a broader population-based strategy capable of protecting most infants during the period of higher susceptibility to severe disease (6, 7, 10, 99, 101, 103, 133). These interventions have the potential not only to reduce RSV-related hospitalizations and healthcare utilization, but also to mitigate disparities in respiratory health and possibly influence long-term respiratory outcomes (30, 91, 92, 94).

Importantly, the lessons learned from RSV extend beyond a single pathogen. The identification of conserved viral epitopes, the development of long-acting immunoprophylaxis, and the integration of passive and active immunization strategies provide a framework for addressing other major pediatric respiratory viruses (Table 1). For influenza and SARS-CoV-2, targeting conserved structures, improving vaccine adaptability, and maintaining continuous genomic surveillance will be essential to overcome antigenic evolution and immune escape (11, 126, 139, 161, 162) (Table 1). For emerging targets such as rhinovirus, human metapneumovirus, and parainfluenza viruses, early evidence suggests that broadly neutralizing antibodies and next-generation vaccine platforms may help address longstanding barriers to prevention (13, 156, 166, 199) (Table 1).

**Table 1:** Current status of vaccines and monoclonal antibodies for major pediatric respiratory viruses.

Virus	Approved vaccines	Approved mAbs	Pipeline vaccines	Pipeline mAbs	Main challenges	Future directions
RSV	Maternal preF vaccine	Nirsevimab Clesrovimab	Next-generation vaccines	Extended half-life mAbs	Global access	Combination active/passive strategies
Influenza	Seasonal vaccines	None approved for prevention	Universal vaccines	Broadly neutralizing anti-HA mAbs	Antigenic drift/shift	Conserved HA stem targeting
SARS-CoV-2	mRNA/protein vaccines	Variant-dependent therapeutic mAbs	Pan-coronavirus vaccines	Broad-spectrum mAbs	Rapid variant emergence	Conserved spike targets
HMPV	None	None	F-based vaccines	Anti-F mAbs	Limited pediatric data	Shared RSV/HMPV epitopes
PI	None	None	Lieve-attenuated/vector vaccines	Anti-F mAbs	Limited immunogenicity	Conserved fusion targets
Rh	None	None	Multivalent vaccines	Experimental receptor-targeted mAbs	Extreme antigenic diversity	Multivalent platforms

RSV, respiratory syncytial virus; SARS-CoV-2, severe acute respiratory syndrome coronavirus 2; HMPV, human metapneumovirus; PI, parainfluenza; Rh, rhinovirus; mAb, monoclonal antibody; F, fusion protein; HA, hemagglutinin.

Future efforts should focus on optimizing preventive strategies, generating robust real-world pediatric evidence, and ensuring equitable global access to these innovations. Applying the lessons learned from RSV will be critical to achieving more effective, durable, and comprehensive protection against respiratory viral infections in children worldwide (13, 156, 166, 199).
